# Characterization of a New Series of Fluorescent Probes for Imaging Membrane Order

**DOI:** 10.1371/journal.pone.0052960

**Published:** 2013-02-04

**Authors:** Joanna M. Kwiatek, Dylan M. Owen, Ahmed Abu-Siniyeh, Ping Yan, Leslie M. Loew, Katharina Gaus

**Affiliations:** 1 Centre for Vascular Research and Australian Centre for Nanomedicine, University of New South Wales, Sydney, Australia; 2 Center for Cell Analysis and Modelling, University of Connecticut Health Center, Farmington, Connecticut, United States of America; Institut Curie, France

## Abstract

Visualization and quantification of lipid order is an important tool in membrane biophysics and cell biology, but the availability of environmentally sensitive fluorescent membrane probes is limited. Here, we present the characterization of the novel fluorescent dyes PY3304, PY3174 and PY3184, whose fluorescence properties are sensitive to membrane lipid order. In artificial bilayers, the fluorescence emission spectra are red-shifted between the liquid-ordered and liquid-disordered phases. Using ratiometric imaging we demonstrate that the degree of membrane order can be quantitatively determined in artificial liposomes as well as live cells and intact, live zebrafish embryos. Finally, we show that the fluorescence lifetime of the dyes is also dependent on bilayer order. These probes expand the current palate of lipid order-sensing fluorophores affording greater flexibility in the excitation/emission wavelengths and possibly new opportunities in membrane biology.

## Introduction

Lipids and proteins are not homogenously distributed in the plasma membrane of eukaryotic cells giving raise to biochemically and biophysically distinct domains. In artificial bilayers, the membrane can separate into immiscible liquid-ordered and liquid-disordered phases that differ in the degree of lipid packing. Highly ordered domains, known as lipid rafts, have been postulated to also exist in the cell membrane with implications for protein distributions and diffusion [1], [2], [3], [4]. Numerous cellular processes including receptor signaling, for example at the immunological synapse of T cells [5], [6], and membrane trafficking [7], for example in polarized epithelial cells 8,9] are thought to be influenced by membrane order. Lipid domains with a high membrane order have also been shown to play a role in a number of pathologies [10], [11].

Fluorescence microscopy is the tool-of-choice to investigate membrane lipid domains [12], [13] as it can equally be applied to model and cell membranes and avoids fixation, which can introduce artifacts in lipid and protein organization [14]. The properties of lipid domains can be investigated by analyzing the localization and dynamics of membrane proteins and lipids [15], however, most of these approaches rely on prior knowledge of the preference of the probe for partitioning into the ordered and disordered domains. A more direct approach is the use of environmentally sensitive membrane dyes [5], [16], [17], [18], [19], [20]. These probes typically change their fluorescence properties based on the polarity of their local solvent [21]. Since the degree of lipid packing changes the dyes' local molecular environment, liquid-ordered and liquid-disordered membranes can be distinguished by the differential penetration of polar water molecules into the otherwise non-polar bilayer interior.

One of the most popular polarity-sensitive dyes for the investigation of membrane organization is Laurdan (6-lauryl-2-dimethylamino-naphthalene), which is a derivative of Prodan created by Weber and Farris in 1979 [22]. Laurdan excites at ∼400 nm or ∼800 nm using multiphoton excitation and displays a 50 nm red-shift between the ordered phases composed of PSM: Chol 7∶3 (n-palmitoyl-sphingomyelin:cholesterol 7∶3; emission peak ∼460 nm) and disordered phases of 100% DOPC (1,2-dioleoyl-*sn*-glycero-3-phosphocholine; emission peak ∼510 nm) phases. Hence membrane order can be quantified by 2-channel ratiometric imaging [18]. Laurdan can be used to stain artificial membranes, live and fixed cells, as well as whole organisms [9] without any adverse effects on the membranes under investigation [18]. Despite its utility, Laurdan suffers from considerable drawbacks because UV illumination of live cells can result in phototoxic effects. It should be noted that single-photon UV excitation of Laurdan is possible which then enables widefield imaging techniques such as total internal reflection fluorescence (TIRF) [23]. While overcoming many of the drawbacks of UV excitation, multi-photon excitation requires complex and expensive pulsed lasers [17], [24], which are not available in many laboratories. A related probe is C-Laurdan (6-dodecanoyl-2-[N-methyl-N-(carboxymethyl)amino]naphthalene), which has higher solubility in aqueous media than Laurdan and is more photostable. However, the dye still excites around 400 nm and therefore requires the use of UV or multi-photon excitation [25]. An alternative probe is di-4-ANEPPDHQ which was designed as a voltage-sensitive dye to monitor electrical activity in cells and tissues [26] but can be also used to quantify membrane order [5], [20], [27]. Unlike Laurdan, di-4-ANEPPDHQ is excited at 488 nm and presents a 60 nm red shift between liquid-ordered (PSM:Chol 7∶3; emission peak ∼560 nm) and liquid-disordered (100% DOPC; emission peak ∼620 nm) phases. This makes it amenable to confocal microscopy using single-photon excitation as well as TIRF microscopy and flow cytometry [5], [28]. However, its broad emission spectrum in the green-red region makes multi-labeling challenging. Hence, there is a need of new polarity-sensitive probes that may offer greater flexibility for multi-modal imaging.

**Figure 1:**
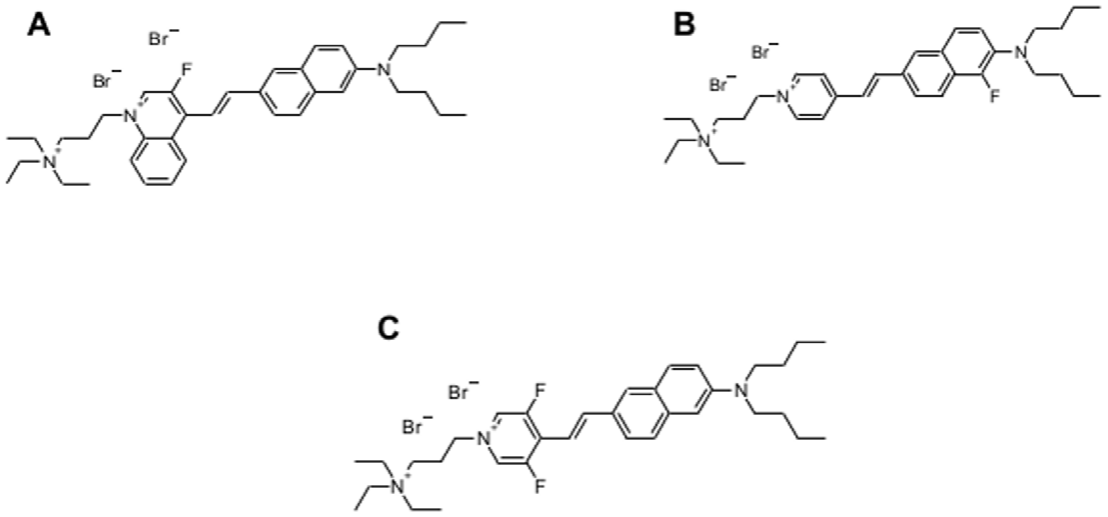
Structures of fluorescent probes. The structure of PY3304 (A), PY3174 (B), PY3184 (C).

Due to the above limitations, designing new membrane probes is an area of ongoing research [29]. The purpose of this study is to test a series of new probes (Figure 1) for their ability to sense lipid order in model and cell membranes. The dyes demonstrated here are PY3304 (di-4-ANEQ(F)PTEA; 4-[2-(6-Dibutylamino-naphthalen-2-yl)-vinyl]-1-(3-triiethylammonio-propyl)-3-fluoro-quinolinium dibromide), PY3174 (di-4-AN(F)EPPTEA; 4-[2-(6-Dibutylamino-5-fluoro-naphthalen-2-yl)-vinyl]-1-(3-triethylammonio-propyl)-pyridinium dibromide) and PY3184 (di-4-ANEP(F2)PTEA; 4-[2-(6-Dibutylamino-naphthalen-2-yl)-vinyl]-1-(3-triethylammonio-propyl)-3,5-difluoro-pyridinium dibromide. Throughout the manuscript, we refer to the extent of lateral packing as lipid order, which in case of Laurdan is inferred from solvent dipolar relaxation. Although the precise spectroscopic properties of the new dyes has not yet been determined in detail, the study was designed to provide further motivation for the design and synthesis of new membrane probes and accelerate the uptake of such dyes by the biological community. The dyes were chosen for this study because of their excitation and emission properties that may enable unique combinations for multi-labeling and multi-modal imaging, thereby expanding the palate of lipid order-sensitive fluorophores for the quantitative analysis of membrane order.

Our data show that the three new dyes are characterized by red-shifted emission spectra between the liquid-ordered and liquid-disordered phases. Moreover, we demonstrate that the dyes can be used in live cells and in whole zebrafish embryos where differences in membrane order between cellular compartments can be identified. Critically, these dyes show a range of excitation and emission profiles allowing the choice of fluorophore to be tailored to the desired experiment. For example, short excitation wavelength dyes can be used to match available laser lines, or longer excitation wavelengths used to allow deeper penetration in to tissue or for multiplexing with other molecular markers such as fluorescent proteins.

## Materials and Methods

Artificial disordered and ordered phase bilayers (small unilamellar vesicles, SUVs or giant unilamellar vesicles, GUVs) were prepared with 1,2-dioleoyl-sn-glycer-3-phosphocholine (DOPC) and a 7∶3 mixture of n-palmitoyl-sphingomyelin (PSM) and cholesterol (all Avanti Polar Lipids), respectively [30]. GUVs which exhibit coexisting phases were formed from a composition of 1∶1∶1 DOPC:PSM:Cholesterol. Vesicles were incubated for 15 min at room temperature with PY3304, PY3174 or PY3184 in water at 20–30 μM and imaged at room temperature.

HeLa cells were cultured in Dulbecco's minimum essential medium (DMEM) with 10% fetal calf serum (FCS) at 37°C in a 5% CO_2_ atmosphere. For microscopy, cells were plated in glass-bottom microscope dishes and prior to imaging were washed three times with phosphate buffered saline (PBS) and labeled with PY3304, PY3174 or PY3184 in DMEM for 30 min at 5–10 μM. Cells were imaged at room temperature. The 30 min incubation times allows for the dye to internalize and equilibrate between the different cellular membranes. Cell viability after incubating HeLa cells with 10 µm PY3304, PY3174 or PY3184 for 40 min was similar to control cells, as determined by lactate dehydrogenase (LDH) release into media.

Three day-old zebrafish embryos were incubated with dyes PY3304, PY3174 or PY3184 (2 µM −5 ) dissolved in E3 media for 2 h. The embryos were anesthetized with 50 µL of 0.04% (wt/vol) tricaine and embedded into low-melting agarose gel in a glass-bottom microscope dish as previously described [9].

Emission spectra of PY3304, PY3174 and PY3184 in artificial bilayers were obtained using a fluorescence spectrophotometer (Cary Eclipse, Agilent Technologies). Spectra were recorded in the excitation range 300–750 nm with 10 nm spectral resolution and between 400–1000 nm with 10 nm resolution for fluorescence emission.

Images of artificial bilayers and live cells were acquired using a confocal fluorescence microscope (TCS SP5, Leica Microsystems) with a 1.2NA, 63X water-immersion objective. PY3304 was excited at 561 nm, PY3174 and PY3184 were excited at 488 nm. These wavelengths were chosen because they are close to the peak excitation in each case and are laser lines that are common on most microscopy systems. Zebrafish images were acquired using a 1.3 NA, 63X glycerol-immersion objective using femtosecond-pulsed titanium-sapphire laser (Mai-Tai, Spectra-Physics) for multiphoton excitation (1040 nm for PY3304, 900 nm for PY3174 and 1000 nm for PY3184). 2–channel fluorescence emission was acquired in the following ranges: 570–640 nm and 655–750 nm for PY3304; 505–590 nm and 620–690 nm for PY3174; 510–610 nm and 630–700 nm for PY3184.

Generalized Polarization [24] (GP) images were calculated using a custom plugin for ImageJ [18] from the intensity of the ordered phase image (I_O_, shorter wavelength) and a red-shifted image (I_D_) from the disordered phase according to Equation 1 [18]:
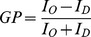
(1)


The GP values range from −1 (when all the fluorescence emission is collected in the disordered, long-wavelength channel) to +1 (when all the fluorescence is collected in the ordered, short wavelength channel). The GP value is thus a measure of membrane order. To describe the ‘amount’ of contrast between the ordered and disordered membranes in the image we calculate a figure of merit (FoM) calculated using Equation 2:
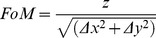
(2)where *z* describes distance between peaks of the histograms of the GP values for ordered and disordered membranes and *Δx* and *Δy* are the histogram widths respectively.

Fluorescence lifetime imaging (FLIM) was performed using time-correlated single-photon counting (TCSPC) (Microtime200, Picoquant GmbH). PY3304 was excited using picosecond pulsed illumination at 532 nm and fluorescence collected using a 550 nm long-pass filter. PY3174 and PY3184 were excited at 473 nm and fluorescence detected using a 550 nm long-pass filter. Detection occurred by a single-photon avalanche diode (SPAD). Fluorescence decays were fitted to single decay functions and color-coded according to mean lifetime.

## Results and Discussion

**Figure 2:**
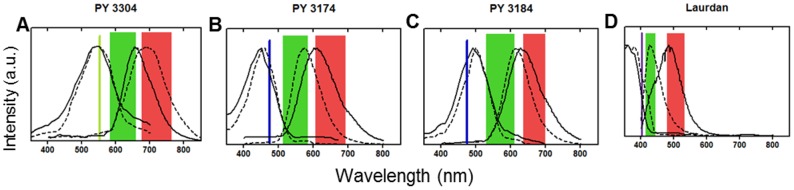
Excitation and emission spectra of artificial bilayers. The wavelength bands for 2-channel acquisitions are indicated by shaded boxes. Dashed line corresponds to liquid ordered phase and continuous line corresponds to liquid disordered phase. The solid vertical line corresponds to the excitation wavelength used for microscopy experiments.

We tested the performance of three new dyes, PY3304, PY3174 and PY3184 as membrane order-sensing probes. All of the dyes exhibit negligible fluorescence in aqueous media but strong fluorescence when incorporated into membranes (data not shown). The structures of the three related probes are shown in Figure 1 A, B, C. We first measured the excitation and emission spectra of the three dyes incorporated into artificial membranes with homogenous liquid-ordered and disordered phases (Fig. 2). From these spectra, the wavelengths corresponding to peak fluorescence emission were: 540 nm for PY3304 (Fig. 2A), 440 nm for PY3174 (Fig. 2B) and 490 nm for PY3184 (Fig. 2C). Plots were normalized to the total number of collected photons. The excitation spectra show that the probes can be excited using the common laser lines 561 nm (PY3304) and 488 nm (PY3174 and PY3184), respectively. The fluorescence emission spectra of all three probes show a red-shift between the liquid-ordered and liquid-disordered phases, indicating they could be used to distinguish different lipid phases. PY3304 and PY3174 show the greatest red shift between the two phases of 34 nm and 26 nm, respectively, while PY 3184 shows a 12 nm red shift between liquid ordered and liquid disordered phases. Theses shifts are comparable to the 54 nm red shift obtained for Laurdan in identical membranes (Fig. 2D).

**Figure 3:**
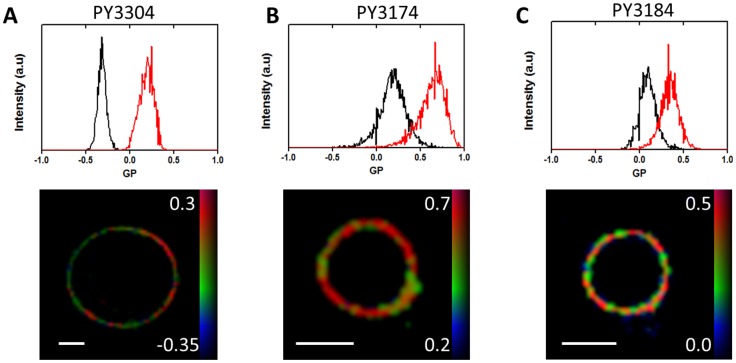
Analysis of Giant Unilamellar Vesicles. Top: Histograms of the GP values obtained from 2-channel confocal images of artificial bilayers in liquid ordered phase (red line) and liquid disordered phase (black line) obtained with PY3304 (A), PY3174 (B), PY3184 (C). Histograms are normalized to the total number of pixels. Bottom: GP images of 1∶1∶1 DOPC:PSM:cholesterol GUVs showing coexistence of ordered and disordered phase membranes stained with the three probes and imaged by 2-channel confocal microscopy. Scale bar  = 5 μm.

From the emission spectra, we selected spectral windows used for ratiometric 2-channel confocal imaging (shaded green and red in Fig. 2, respectively) of artificial membranes stained with the three probes. From these images, we calculated the distribution of GP values, whose histograms are shown in Figure 3 (top row). The average GP values and their standard deviations for liquid-ordered and liquid-disordered phase were 0.111±0.052 and −0.317±0.015 respectively for PY3304; for PY3174 these values were 0.648±0.056 and 0.192±0.028, respectively; and for PY3184, the ordered phase GP value was 0.346±0.023 and the disordered phase GP value was 0.111±0.053. Hence, the greatest difference in GP between the ordered and disordered phases was found for PY3174. From these histograms, a figure of merit (FoM) was calculated to estimate the amount of contrast in membrane order generated by each dye. These values were 1.195 (PY3304), 1.073 (PY3174) and 1.072 (PY3184). Hence, PY3304 gives the maximum image contrast between ordered and disordered membranes. Nevertheless, GP images (Fig 3, bottom row) clearly show that coexisting phases could be distinguished with all three probes in GUVS that contained both ordered (high GP) and disordered (low GP) membrane regions. Measuring the relative incorporation of the dyes into liposomes in ordered and disordered phases revealed that all three dyes have a similar high affinity for bilayers with a slight but not significant preference for the disordered lipid phase (data not shown). Hence, all three new probes efficiently stain both ordered and disordered membranes. In addition, we did not observe any significant photoselection effects with these dyes. This is in contrast to Laurdan were photoselection due to the polarization of the excitation light is observed (data not shown). This may indicate that the new probes are held in a less rigid configuration within the lipid bilayer.

**Figure 4:**
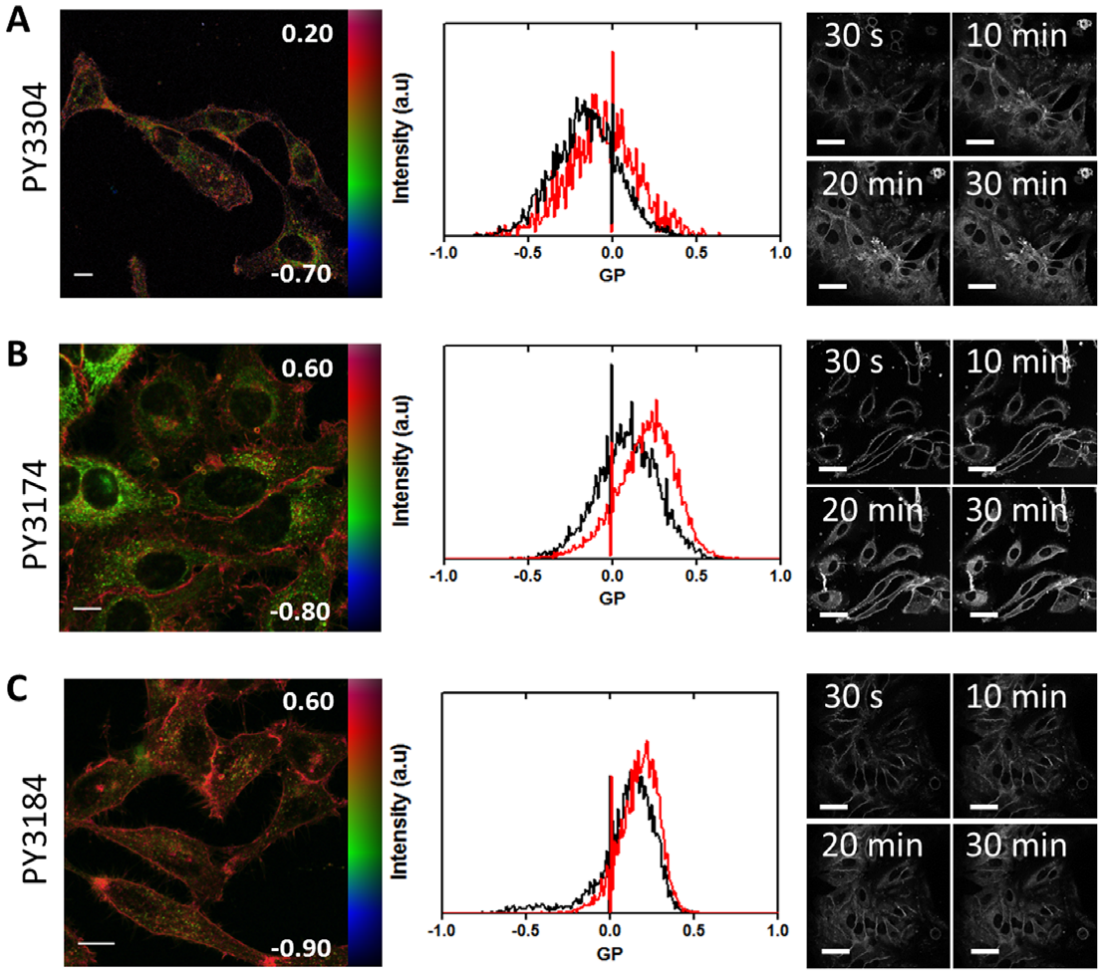
Analysis of live HeLa cells. Left: GP images of live HeLa cells stained with PY3304 (A), PY3174 (B), PY3184 (C). GP images are in false color and run over the range indicated by the color bars to indicate a higher degree of membrane order (predominately colored red) in the plasma membrane compared to intracellular membranes (predominately colored green). Scale bars 10 µm. Middle: Histograms of the GP values obtained from GP images of live HeLa cells stained with PY3304 (A), PY3174 (B), PY3184 (C). Histograms obtained from ROIs for plasma membrane (red line) and intracellular membranes (black) were normalized to the total number of pixels. Right: Staining profile of Live HeLa cells. HeLa cells were incubated with PY3304 (A), PY3174 (B) and PY3184 (C) for 30 min. Confocal intensity images were acquired at 30 s, 10 min, 20 min, and 30 min after dyes were added. Images show complete staining of all cellular membranes after 30 min. Scale bars 50 µm.

To demonstrate that the dyes can be used to quantify membrane order in cells, we imaged live HeLa cells and calculated GP values from the plasma membrane (PM) and intracellular membranes (IM) (Figure 4). Representative pseudo-colored GP images of the cells and the associated GP histograms from those images are shown in Fig. 4A, B, C. Figure 4 also shows a time course of staining for each dye indicating that all membranes are stained within 30 minutes. From the GP images it can clearly be seen that the PM has higher GP values than the IM for all dyes indicating high membrane order in the plasma membrane as previously observed for other dyes [18]. It was also noticed that the internalization rate and hence the staining of IM was not identical for all dyes. For PY3304, GP values were −0.061±0.038 and −0.161±0.053 for the PM and IM, respectively. For PY3174, the PM GP values were 0.204±0.033 and the IM GP values were 0.101±0.048. For PY3184 these values were 0.180±0.018 and 0.142±0.035, respectively. The following FoM values were calculated from these histograms: 0.222 (PY3304), 0.217 (PY3174) and 0.208 (PY3184). As expected from the characterization in model membranes, the PY3304 gave the highest contrast in GP ratiometric imaging.

**Figure 5:**
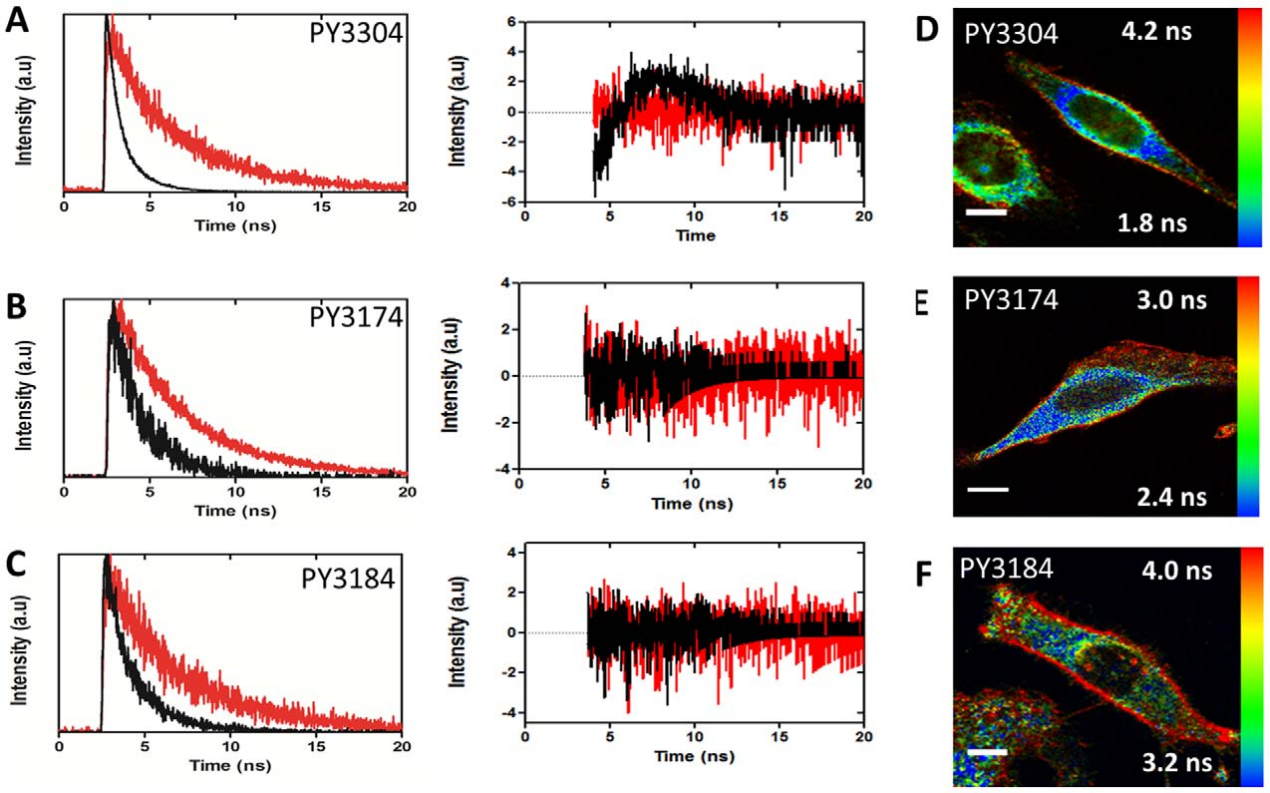
Analysis of FLIM images. Left: Fluorescence decay histograms acquired from artificial membranes stained with PY3304 (A), PY3174 (B) and PY3184 (C) showing longer lifetimes in ordered membranes (red) than disordered membranes (black). Middle: Plots of residuals from fitting fluorescence decay histograms. (D-F) Right: Fluorescence lifetime images of live HeLa cells stained with PY3304 (D), PY3174 (E), PY3184 (F). Images show an increased order at the plasma membrane in agreement with the spectral measurements and previously published results. Scale bar  = 10 μm.

As well as spectral imaging, fluorescence lifetime has also been demonstrated for imaging membrane order with several advantages [31], [32]. Representative ordered and disordered lifetime decays and residuals from a single exponential fit acquired from artificial membranes are shown in Fig. 5A, B, C. In artificial bilayers, we measured fluorescence lifetimes for the liquid-ordered and liquid-disordered phases of τ = 4.1 ns and τ = 1.0 ns respectively for PY3304. For PY3174 these values were τ = 4.4 ns and τ = 1.7 ns respectively. For PY3184, the ordered phase lifetime value was 4.3 ns and the disordered phase lifetime value was 1.8 ns. Higher fluorescence lifetimes in the ordered than disordered phase have been reported previously for related dyes [31]. Figure 5D, E, F show fluorescence lifetime images of each dye acquired in live HeLa cells where long lifetimes (more densely packed bilayers) were pseudo-colored red and disordered phase (less densely packed bilayers) were colored blue. Again, differences between the plasma membrane and internal membranes are easily detected with all three probes.

**Figure 6:**
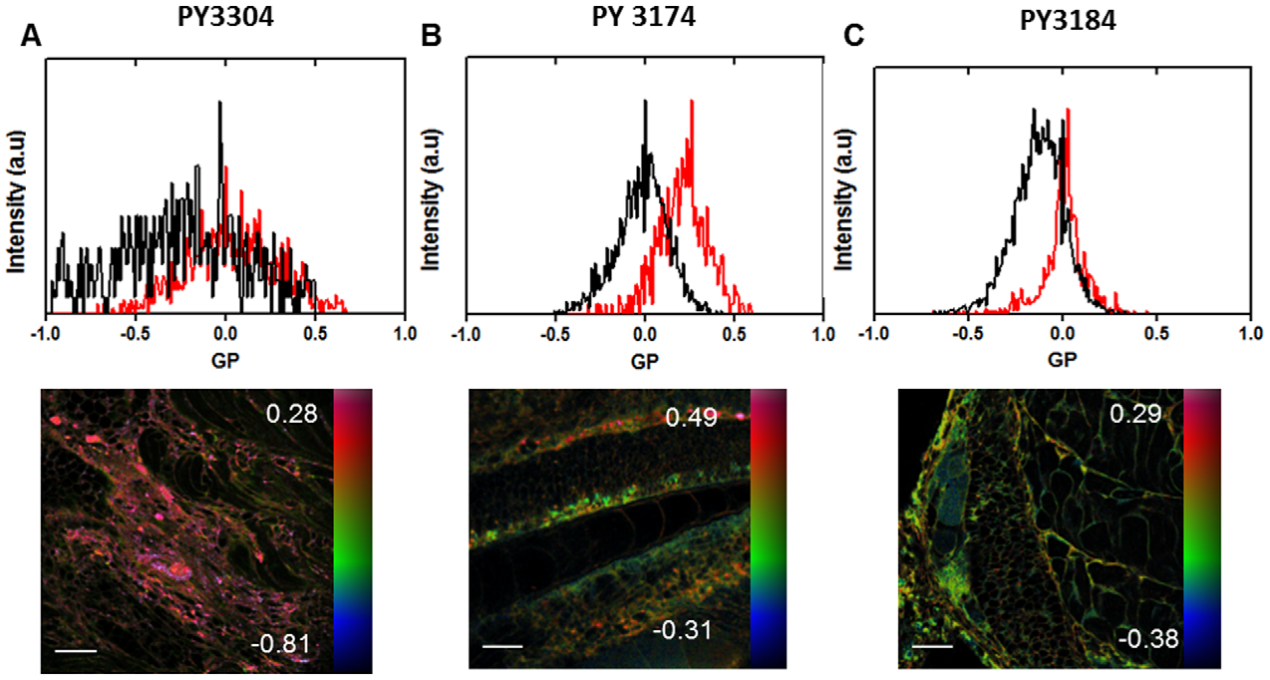
Analysis of live Zebrafish embryos. Top: Histograms of the GP values obtained from GP images of live Zebrafish embryos stained with PY3304 (A), PY3174 (B), PY3184 (C). Histograms obtained from ROIs for plasma membrane (red line) and intracellular membranes (black) were normalized to the total number of pixels. Bottom: GP images are in false color and run over the range indicated by the color bars to indicate a higher degree of membrane order (predominately colored red) in the plasma membrane compared to intracellular membranes (predominately colored green). Embryos were stained with PY3304 (left), PY3174 (middle), PY3184 (right) excited by multi-photon excitation at 1040 nm for PY3304, 900 nm for PY 3174, 1000 nm for PY 3184. Scale bars  = 10 µm.

Finally, we demonstrate that the three dyes can be used to stain and image membrane order in intact, live zebrafish embryos. This has been previously demonstrated using Laurdan and multiphoton microscopy [9]. Here, the longer excitation wavelengths of the novel probes are advantageous when used with multiphoton excitation as it allows deeper imaging through tissue. Figure 6 shows GP histograms (top row) and GP images (bottom row) of live Zebrafish embryos stained with PY3304 (A), PY3174 (B) and PY3184 (C). From these images we calculated an average GP value for regions-of-interest corresponding to the plasma membrane and intracellular membranes. The values for these regions were 0.021±0.039 and −0.165±0.064 for the plasma membrane and intracellular membranes, respectively for PY3304. For PY3174 the values were 0.224±0.081 and −0.001±0.050, respectively; and for PY3184, the plasma membrane was 0.030±0.032 and intracellular membranes were −0.129±0.037. The following FoM values were calculated from the histograms: 0.320 (PY3304), 0.600 (PY3174) and 0.626 (PY3184). The images illustrate that all three dyes can be used for multi-photon imaging in intact tissue.

## Conclusion

Membrane order is an important biophysical characteristic that is thought to regulate many cellular membrane processes [4]. It is possible to image the distribution of membrane order using environmentally sensitive fluorophores such as Laurdan [17] and di-4-ANEPPDHQ [20]. In this article we present a new series of order-sensitive optical probes, PY3304, PY3174 and PY3184 for analyzing membrane order in artificial membranes, live cells and live, intact vertebrate organisms. The presented probes are polarity sensitive and can be excited with single or multiphoton laser illumination. For all three dyes, fluorescence emission in the disordered phase is red-shifted and displays a shorter fluorescence lifetime than fluorescence from the ordered phase. This allows membrane order to be imaged by 2-channel ratiometric (GP) imaging or via single-channel fluorescence lifetime imaging. In both cases, PY3304 gave the greatest contrast between the liquid-disordered and liquid-ordered phases.

The GP formulation was originally used for Laurdan based on the observation that Laurdan exhibits different degrees of solvent relaxation in gel and liquid-disordered phases in glycerophospholipid membranes [16], [33]. This is equivalent to two ‘states’ and analogous to two different polarization states in fluorescence anisotropy imaging. It is currently not known whether the new PY-series dyes have two corresponding states and a quantitative description of the probe's dipole states should not be inferred from our GP measurements. Nevertheless, our data clearly show that the GP function can be used to test the suitability of new dyes as membrane reporters and was here used to generate image contrast based on lipid packing. While a detailed characterization of each probes photo-physical properties and response mechanism(s) is an important area of future investigations, our results show that these dyes can distinguish between the classical liquid-ordered and liquid-disordered phases in model membrane and regions of higher and lower order in cell membranes.

It should be noted that, like Laurdan, these probes are likely to have their dipole transition moment orientated perpendicular to the plane of the bilayer. As demonstrated for Laurdan [34], this precludes excitation in the gel phase when the membrane is orientated parallel to the excitation polarization. This may result in erroneous GP values in this geometry due to the preferential excitation of liquid-disordered-localized dye, which has greater orientational freedom. Given the similar dipole orientation, this is a limitation that these new dyes are unlikely to overcome.

We have shown that the new PY-series dyes report differences in the degree of lateral membrane lipid packing in live cells and in tissues of intact zebrafish embryos. Similar observations have been reported for Laurdan and di-4-ANEPPDHQ [17], [18], [20]. There is an ongoing debate how to interpret such heterogeneities given that lipid rafts are partially defined by exactly this biophysical property. However, due to the resolution of diffraction-limited imaging, it should be noted that regions of higher lipid order do not represent individual lipid raft domains. The GP value of each pixel is an average over this resolution element that may contain distinct lipid domains of various degree of lipid packing density, size and abundance.

In conclusion, the study has extended the palate of available fluorophores for the quantitative imaging of lipid order in model and cell membranes. Each dye presented here has a unique combination of excitation and emission properties. The range of excitation and emission wavelengths allows the choice of dye to be tailored to a particular experiment. For example, the dyes can be matched to available laser lines or to multiplexing with other fluorophores such as fluorescent proteins or small molecule dyes. Further, some dyes show red-shifted emission spectra, which when combined with multi-photon excitation, allows deeper imaging into tissue for potential *in vivo* studies of membrane order.
